# Micronutrients in Food Supplements for Pregnant Women: European Health Claims Assessment

**DOI:** 10.3390/nu15214592

**Published:** 2023-10-28

**Authors:** Laura Domínguez, Virginia Fernández-Ruiz, Montaña Cámara

**Affiliations:** Nutrition and Food Science Department, Faculty of Pharmacy, Complutense University of Madrid (UCM), Plaza Ramón y Cajal, s/n, E-28040 Madrid, Spain; vfernand@ucm.es (V.F.-R.); mcamara@ucm.es (M.C.)

**Keywords:** micronutrient, food supplement, health claim, labeling, European legislation

## Abstract

Micronutrients play a critical role in pregnant women, a vulnerable group with higher nutritional requirements. The first strategy to achieve adequate micronutrients intake should always be through a healthy and balanced diet. In the case where the diet is not enough to meet these requirements, food supplements should be prescribed under supervision to complement the diet, and these products must bear reliable information about the declared nutritional contents and health benefits. Based on the data provided by the Coordinated System of Fast Interchange of Information (SCIRI) and to know the current national situation, this work addresses the assessment of the content and the adequacy of health claims related to some micronutrients (vitamin C, vitamin B_9_, iron, copper, manganese, zinc, calcium, magnesium) contained in food supplements for pregnant women commercialized in Spain. Analytical results coincided with the declared values and were covered by the ranges of tolerances, and samples met the requirements to use health claims. Although the samples could even include more claims, manufacturers could have selected those which either best addressed pregnant women’s conditions or best aligned with marketing intentions. This study confirms an adequate use of health claims in food supplement samples, which could be interesting for strengthening consumers’ confidence in the benefits shown in the labeling and for encouraging the use of health claims as a useful tool for making better-informed purchasing decisions.

## 1. Introduction

Food supplements are concentrated sources of micronutrients (vitamins, minerals) with a nutritional and positive physiological effect that are commercialized in different dose forms (e.g., capsules, pills, sachets of powder, etc.) [1,2,3], and they are intended to complement diet, improve health status, or even reduce the risk of some diseases [4,5,6].

In 2022, a survey conducted in 14 European Member States, including Spain, revealed that almost 9 in 10 European consumers had taken food supplements in their lives, and the great majority of them (93%) had done it in the past year [7]. Baladia et al. (2021) performed a comprehensive market study on the consumption of these products by the Spanish population (18–65 years), and the results demonstrated that 70% of the studied Spanish sample (N = 2630) took at least one food supplement during a specific period in 2020, with the most used (63%) being the ones containing vitamins and minerals. Vitamin C (31%) and magnesium, calcium, iron, zinc, and copper (6–13%) are the most frequently present vitamins and minerals in these food supplements [8].

Pregnant women are a vulnerable group of population whose nutritional requirements are high. The diet before and during pregnancy is a crucial element for the optimal development and growth of the fetus, as the nutritional status of pregnant women directly influences the size of the placenta, which is the main connection link with the fetus, as well as cell proliferation and differentiation [9,10,11,12].

A healthy diet is assumed to cover all nutritional needs; however, sometimes, diets are not very well equilibrated to provide enough amounts of some micronutrients such as vitamins (folic acid; vitamins C, D, B_1_, B_2_, B_6_) and minerals (calcium, magnesium, iron, zinc, copper, manganese, etc.) with important health benefits. Thus, their daily intake must be reinforced through supplementation to avoid micronutrient deficiencies [9,10,11,12,13,14].

Folic acid is probably the most crucial micronutrient before and during pregnancy. Folic acid deficiency is related to the appearance of anemia, complications during pregnancy, and serious health problems in the fetus such as intrauterine growth restriction, premature birth, low birth weight, neural tube defects, and other fetal malformations [15,16]. Neural tube defects are the most common congenital anomalies that cause severe disability and infant mortality. Up till now, scientific evidence has shown that an adequate folic acid supplementation (≥400 μg/day) before and during the first weeks of pregnancy can reduce the risk of neural tube defects in the fetus in up to 46% of cases [9,10].

Iron deficiency is the most common micronutrient deficiency during pregnancy that provokes anemia [17]. According to the World Health Organization (WHO), 40% of pregnant women around the world suffer anemia because of an inappropriate daily intake of this mineral. Iron supplementation during pregnancy is, therefore, considered essential [14]. Calcium is another fundamental mineral as its deficiency can cause a low birth weight and negatively affect bone mineral content in children [13]. In addition, low calcium intake is associated with the onset of preeclampsia, a pregnancy-specific multi-systemic disorder that provokes hypertension and proteinuria in pregnant women, and it is considered a leading cause of maternal mortality globally [18,19]. Antenatal calcium supplementation is also recommended by the WHO as it can prevent approximately one-half of preeclampsia cases in accordance with the scientific literature [14]. Zinc and copper are also considered indispensable micronutrients during pregnancy. A moderate maternal zinc deficiency may cause alterations in protein synthesis and cellular replication, which could affect fetal development and lead to malformations and a low birth weight [13,20]. Low serum levels of copper in pregnant women are linked to a premature rupture of membranes, weak amniotic membrane, and spontaneous abortion [21]. Turan et al. (2019) observed that miscarriage rates were significantly higher in pregnant women with lower serum copper concentrations (30–35%) [22], whereas optimum levels of this mineral at 15 weeks of gestation could reduce spontaneous abortion [23]. Maternal supplementation with vitamin C has been widely studied to elucidate its potential health benefits as it has been suggested that it could decrease the risk of appearance of maternal anemia and other complications such as preeclampsia and intrauterine growth restriction [24,25]. Along with iron, zinc, and copper, vitamin C is included in the United Nations International Multiple Micronutrient Antenatal Preparation (UNIMMAP), an established formulation that is recommended by the WHO and contains 15 micronutrients (vitamins and minerals) to provide pregnant women and their offspring with a healthy start to life [14]. Regarding magnesium, scientific evidence suggests that this mineral has a significant role in glucose metabolism, and low serum concentrations have been associated with gestational diabetes mellitus. This mineral could also have a protective role against pregnancy inflammation through the inhibition of nitric oxide synthase enzyme [26].

Apart from these micronutrients, there are other important vitamins needed during pregnancy. Vitamin D is considered to play an important role in bone metabolism through calcium regulating and maintaining phosphate homeostasis. Vitamin D deficiency in pregnant women, above all during the winter months, could increase the risk of preeclampsia, gestational diabetes mellitus, and preterm birth, among others [27,28]. To avoid this deficiency, the WHO and FAO recommend a vitamin D intake of 5 μg (200 IU) per day for pregnant women [27]. Vitamins B_1_, B_2_, and B_6_ are important in different functions of the nervous system. During pregnancy, these vitamins could contribute to the development of fetus brain and nerves. Likewise, vitamins B_2_ and B_6_ could reduce the risk of developing preeclampsia and prevent a low birth weight, respectively [29,30,31].

Pregnant women must be adequately informed about the beneficial effects provided by the consumption of food supplements containing the previously mentioned micronutrients. To protect this vulnerable group of population from misunderstandings and misinformation, health claims included in the labeling of these products must be based on strong scientific evidence and be clear and comprehensible. Thus, pregnant women can rely on those health benefits and make well-informed choices [6,32].

In order to guarantee a constant vigilance of any risk potentially associated with food supplements that could affect the health status of consumers, including pregnant women, Spain counts on with the Coordinated System of Fast Interchange of Information (SCIRI). In a period of seven years (2015–2021), the national network SCIRI reported several notifications regarding food supplements, and most of them were due to an incorrect labeling, including health claims. In fact, in 2021, the notifications related to food supplements were 11 times higher than in 2015 [33].

Based on these alarming data provided by SCIRI reports and with a view to having a clear overview of the current national situation, the objective of the present work is to assess the content and the adequacy of health claims related to micronutrients of food supplements for pregnant women commercialized in the Spanish market according to the European regulation. A prior literature search was carried out by the authors in order to know the state of the art about this matter. To the authors’ knowledge, no scientific articles regarding the evaluation of health claims related to certain micronutrients in food supplements for pregnant women were found. Thus, this study could be useful to widen the knowledge in this particular research perspective, which combines two different areas that are narrowly interconnected (chemical analysis and legislation).

## 2. Materials and Methods

In order to verify the adequation of information regarding health benefits in food supplements for pregnant women, only micronutrients showing health claims in the labeling of these products were considered in this study; that is, vitamin C, vitamin B_9_ (folic acid), iron, copper, manganese, zinc, calcium, and magnesium. Due to the importance of folic acid in pregnancy, the authors published one study in 2021, which was only focused on this micronutrient [6]. With a view to having a more complete overview of this topic, folic acid results are summarized and included in the present work. Other compounds, such as vitamin D, vitamin B_1_, vitamin B_2_, etc., are not addressed in this study and are not chemically analyzed because of the lack of related health claims in the labeling of samples.

### 2.1. Micronutrients’ Health Claims Approved in the European Union (EU)

An updated review of the health claims approved for vitamin C, vitamin B_9_ (folic acid), iron, copper, manganese, zinc, calcium, and magnesium was carried out using the official European Register of nutrition and health claims made on food and food supplements (https://ec.europa.eu/food/safety/labelling_nutrition/claims/register/public/?event=register.home, accessed on 18 June 2023) [34]. The following search filters were used in the European register platform: “claims status: authorised”, “type of claim: Art. 13 and Art. 14(1)(a)”, “EFSA Opinion reference: all”, “Legislation: all”. Use conditions established for each approved health claim were reviewed as well.

### 2.2. Spanish Market Research and Sample Selection

A market research of food supplements for pregnant women with health claims related to micronutrients was completed by consulting 10 national food establishments with a high market share in Spain. In addition, the online purchasing platforms used by these establishments were reviewed to provide a detailed overview of the food supplements offered. Sample selection for analysis was performed through the application of three inclusion criteria: (1) food supplements with a clear and unequivocal indication about the target population (pregnant women) in their labeling; (2) food supplements for pregnant women containing micronutrients in their nutritional composition; and (3) food supplements with health claims related to micronutrients.

### 2.3. Chemical Analysis of Micronutrient Contents in Samples

Vitamin C, folic acid, iron, copper, manganese, zinc, calcium, and magnesium were subject to health claims in food supplement samples; thus, they were chemically analyzed in the laboratory.

#### 2.3.1. Vitamin C: L-Ascorbic Acid

**Figure 1:**
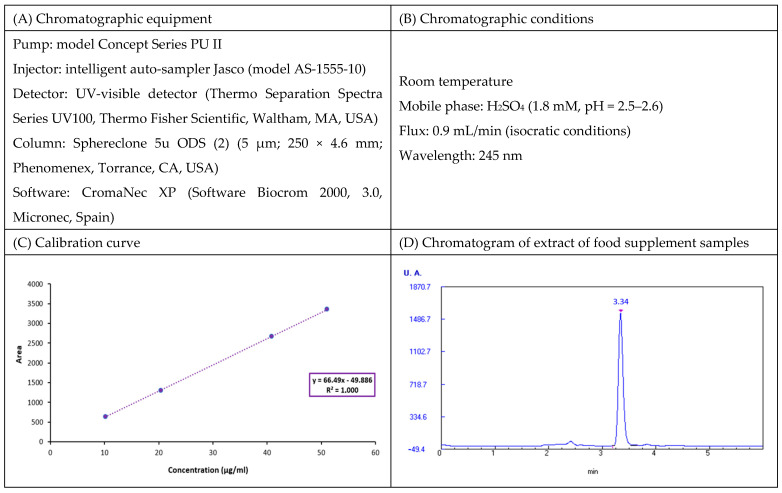
Description of vitamin C determination: (**A**) Chromatographic equipment; (**B**) Chromatographic conditions used in this study; (**C**) Calibration curve; (**D**) Chromatograms obtained for extract of samples containing ascorbic acid through HPLC-UV-visible.

In food matrices, vitamin C can be presented in the forms of L-Ascorbic Acid (AA) (reduced form) and L-Ascorbic Acid Dehydro (DHA) (oxidized form) [35,36]. Although both forms have the same vitamin activity, it is the reduced form that is used commercially [37]. Hence, vitamin C was determined in the form of AA in the food supplement samples. The determination of vitamin C was performed through an extraction in an acid medium, followed by a quantification using a reverse phase in HPLC-UV detection as described by Sánchez-Mata et al. (2000) [38]. An adequate amount of the food supplement samples was weighed, and 25 mL of 4.5% (*w*/*v*) metaphosphoric acid was added. With the aid of a magnetic stirrer (P-Selecta, Asincro), the mixture was shaken for at least 15 min until it dissolved at room temperature and was protected from direct light. The resulting extract was filtered through Albet paper filter No. 1242 (Merck Life Science S.L.U., Madrid, Spain) and collected in an Erlenmeyer flask (Labbox Labware, S.L., Premia de Dalt, Barcelona, Spain). The extract was filtered again with a 0.45 µm Millex PVDF membrane filter (Merck Millipore, Burlington, MA, USA) into a vial and was injected into the chromatographic equipment to quantify it [35]. The calibration curve was plotted daily from a stock solution of AA in metaphosphoric acid, with a final concentration of 0.4 mg/mL. The results were expressed in mg AA/100 g. Figure 1 shows the chromatographic equipment and conditions as well as the calibration curve and one example of the chromatograms obtained for vitamin C analysis.

#### 2.3.2. Vitamin B_9_ (Folic Acid)

Folic acid content was determined in the selected food supplement samples following the method previously published by the authors, being fully described in Domínguez et al. (2021) [6].

#### 2.3.3. Micro- and Macrominerals

The determination of the micro- and macrominerals was carried out following the AOAC 930.05 procedure [39]. From each food supplement, 0.5 g was weighted in porcelain round-bottomed dishes and was subjected to incineration in a microwave oven (Carbolite Furnaces, model CSF 1100; Neuhausen, Germany) from room temperature to 450 °C until white ashes were obtained. Ashes were gravimetrically quantified [40].

Microminerals (Fe, Cu, Mn, Zn) present in ashes were extracted in an acid mixture prepared with 1 mL of HCl:H_2_O (50% *v*/*v*) and 1 mL of HNO_3_:H_2_O (50% *v*/*v*). The solution was filtered by using an Albert^®^ No. 145 ashless filter (Merck Life Science S.L.U., Madrid, Spain) and collected in a volumetric flask, which was made up to 25 mL with distilled water. The resulting extracts were measured through Atomic Absorption Spectroscopy (AAS) (Analyst 200 Perkin Elmer equipment, Perkin Elmer, Waltham, MA, USA) at the specific wavelength for each mineral and using standard solutions for calibration purposes.

An additional 1/10 (*v*/*v*) dilution was completed in La_2_O_3_ (5.864%, *w*/*v*) for the macrominerals’ determination (Ca, Mg). As in the case of microminerals, these solutions were measured through AAS. The results of the micro- and macrominerals were expressed as mg/100 g [39].

#### 2.3.4. Statistical Analysis

Two batches of each food supplement sample were analyzed in triplicate. Data were statistically assessed by comparing both batches of each sample through Tukey’s HSD Test with a level of statistical significance of α = 0.05. Statgraphics Plus 5.1 software was used for statistical analysis.

**Figure 2:**
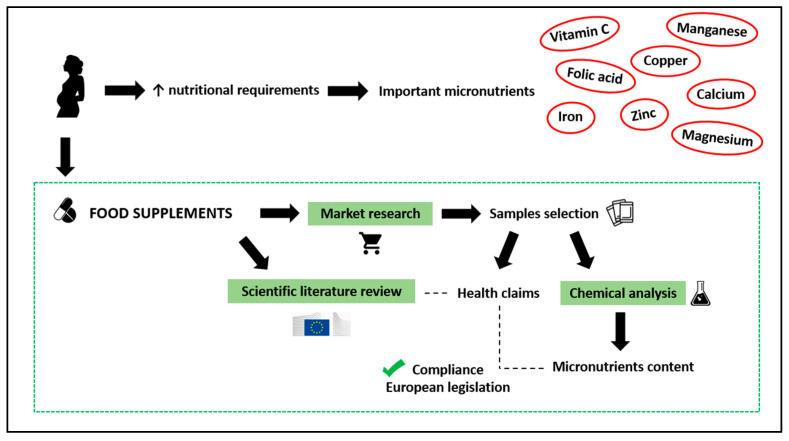
Experimental design to evaluate the adequacy of health claims related to micronutrients of food supplements for pregnant women commercialized in the Spanish market.

For a better understanding, Figure 2 graphically describes the experimental design followed in the present work.

## 3. Results and Discussion

### 3.1. Health Claims Approved in the EU for Analyzed Micronutrients

As shown in Table 1, the micronutrient with the highest number of approved health claims was zinc (18 declarations), followed by vitamin C (15), magnesium (10), copper (8), calcium (8), and iron (7). On the contrary, manganese had the lowest number of authorized health claims (4). Most of the selected micronutrients have been scientifically demonstrated to be able to contribute to a normal energy-yielding metabolism, the immune and nervous systems, as well as to protecting cells from oxidative stress caused by the reactive species of oxygen (ROS) [34].

To use these health claims, food supplements must meet the use conditions particularly established for each nutrient, that is, to be a “Source of [name of vitamin] and/or [name of mineral]”, which means that samples must provide at least 15% of the Nutrient Reference Values (NRVs) of each nutrient supplied by 100 g of the food supplement in question [41,42]. Table 2 provides the minimum content that food supplements must have to include those health claims in their labeling, presentation, and/or advertising.

### 3.2. Spanish Market Search and Samples Selection

A Spanish market search of food supplements for pregnant women containing micronutrients was carried out, with a result of 81 food supplements. The majority (86.5%) were commercialized through online purchasing platforms used by the 10 selected establishments, whereas only 13.5% were found in situ (physical establishments). A total of 4 food supplements met the inclusion criteria, and they were named as the S1, S2, S3, and S4 samples.

### 3.3. Micronutrients Content and Assessment of the Application of Health Claims in Selected Samples

#### 3.3.1. Vitamin C

Only the S1 and S4 samples included ascorbic acid in their formulation. As shown in Table 3, batches 1 and 2 of both food supplements showed similar mean values of vitamin C, and no statistically significant differences were found between batches of the same sample. Total mean value of the S1 and S4 samples coincided with the values included in their composition labeling. According to the European Guidance document published in 2012 by the European Commission regarding the setting of tolerances for nutrient values declared on a label [43], it can be assumed that the analyzed food supplements fulfilled the established requirements as the declared values of ascorbic acid were within the ranges of tolerance (RTs) calculated for each food supplement (Table 3).

Regarding the application of health claims, only the S1 sample showed the following health claim: “Vitamin C contributes to the normal function of the immune system”, although both food supplements contained the minimum amount of vitamin C (12 mg/100 g) required to include in their labeling the 15 health claims approved for this micronutrient.

#### 3.3.2. Vitamin B_9_ (Folic Acid)

As previously reported by the authors [6], all food supplement samples contained folic acid in their nutritional composition. Analytical results of this micronutrient coincided with the declared values in the labeling of samples, and they were covered by the ranges of tolerance (RTs) calculated for each food supplement following the European Guidance document [43] (Table 4).

Regarding health claims, all food supplements (S1, S2, S3, and S4) showed in their labeling at least one health claim approved for folic acid, and they contained more than the 15% NRV (30 µg/100 g) necessary to use them according to the European regulation in force [41,42].

#### 3.3.3. Micro- and Macro-Elements

Regarding the mineral content, mean values of both micro- and macro-elements were similar between batches of the same food supplement samples. No statistically significant differences were found (Table 5).

On the one hand, the Scientific Committee on Food (SCF) and the Panel on Dietetic Products, Nutrition and Allergies (NDA) of the European Food Safety Authority (EFSA) established maximum levels of the total chronic intake of different micronutrients (zinc, calcium, and magnesium, among others) that are unlikely to pose a risk of adverse health effects in humans, which are called tolerable Upper intake Levels (ULs). Different ULs were set according to the age/life-stage group (children, teenagers, adults, pregnant and lactating women). In this case, mean values obtained for zinc, calcium, and magnesium in all food supplement samples were lower than the ULs set for pregnant women (UL zinc = 25 mg/day; UL calcium = 2500 mg/day; UL magnesium = 250 mg/day). No UL had yet been established for iron, copper, and manganese, as sufficient and/or adequate scientific evidence is not available to derive ULs [44,45,46].

On the other hand, to verify if the selected food supplements complied with the requirements established by the European Guidance document, the authors calculated the ranges of tolerances (RT) for those minerals whose content was available in the labeling of each sample. As shown in Table 6, all analytical values were included within the RT.

Regarding health claims, all declarations shown in the labeling of these food supplements fulfilled the strict requirements and use conditions established by the European regulation [41,42].

Focusing on the micro-elements, the S1 sample showed three health claims related to Fe (iron contributes to the normal “formation of red blood cells and haemoglobin” and “function of the immune system”; “iron has a role in the process of cell division”), whereas the S4 sample included only one claim referring to children’s development and health (“iron contributes to normal cognitive development of children”). Both samples could include the seven health claims approved for this mineral as they met the specific use conditions. The S2 and S3 samples did not fulfill these conditions, as their Fe mean values were below the legal limit required to use these health claims (2.1 mg Fe/100 g food supplement).

Only the S1 sample included one health claim referring to Cu in its label (“copper contributes to the normal function of the immune system”), avoiding the use of the other seven authorized health claims. The S2, S3, and S4 samples did not include any Cu health claim, although they could have included a claim due to their Cu content.

Concerning Zn, the labeling of the S1 sample showed 3 health claims (zinc contributes to the normal “fertility and reproduction” and “function of the immune system”; “zinc has a role in the process of cell division”) out of the 18 authorized ones. The S3 sample included only one health claim (“zinc contributes to the normal fertility and reproduction”), whereas the S4 sample did not make any declaration, although its Zn mean value was above the necessary limit to use those claims (1.5 mg Zn/100 g food supplement).

The S3 sample is the only food supplement which included one health claim referring to Mn (“Manganese contributes to the protection of cells from oxidative stress”) out of the four approved ones. The analytical results showed that the S1, S2, and S4 samples could make use of these health claims; however, no Mn claims appeared in their labeling, presentation, and/or advertisement.

Finally, the analytical data obtained for macro-elements in the present study showed that the S1 and S4 samples were able to use 8 and 10 health claims approved for Ca and Mg, respectively, in their labeling. However, the S1 sample included two health claims authorized for Ca (“calcium is needed for the maintenance of normal bones”; “calcium has a role in the process of cell division and specialization”) and one for Mg (“magnesium has a role in the process of cell division”), while the S4 sample only made one claim referring to children’s development and health in relation to the Ca content (“calcium is needed for normal growth and development of bone in children”).

## 4. Conclusions

Reliable information about the declared nutritional contents and the benefits promised by the health claims shown in the labeling of food supplements with micronutrients is crucial for vulnerable groups of the population, such as pregnant women. All analytical results of micronutrients (vitamin C, vitamin B_9_, iron, copper, manganese, zinc, calcium, and/or magnesium) contained in the food supplements selected from the Spanish market coincided with the declared values in their labeling and were within the ranges of tolerance (RTs) established by the European Guidance document published by the European Commission. The zinc, calcium, and magnesium contents in samples were lower than the tolerable upper intake levels established for pregnant women in accordance with scientific deliberations of the Scientific Committee on Food and the EFSA Panel on Dietetic Products, Nutrition and Allergies. In addition, the food supplements analyzed in the present study met all the specific requirements for using the health claims shown in their labeling. Although the samples could use more health claims than the ones already included in their labeling, the manufacturers could have selected those health claims which either best addressed the physiological conditions of this group of population or which best aligned with marketing intentions. Although it would have been interesting to address the chemical analysis of other compounds in the laboratory, this study could be useful to verify the adequate use of health claims related to some micronutrients in the labeling of food supplements as well as to contribute to consumer protection by confirming the inclusion of reliable information about the health benefits provided by the consumption of these products. In addition, the results of the present work could be interesting for strengthening consumers’ confidence, particularly pregnant women, in the benefits shown in the labeling of food supplements and to encourage them to consider these health claims as a useful tool for making better-informed purchasing decisions.

## Figures and Tables

**Table 1:** Approved health claims referring to vitamin C, vitamin B_9_ (folic acid), iron, copper, manganese, zinc, calcium, and magnesium in food supplements according to the European regulation [34].

Micronutrients	Health Claims (N°)	Approved Claims
Vitamin C	15	Vitamin C contributes to maintaining the normal functions of the immune system during and after intense physical exercise; normal collagen formation for the normal function of blood vessels, bones, cartilage, gums, skin, teeth; normal energy-yielding metabolism; functioning of the nervous system; psychological function; function of the immune system; protection of cells from oxidative stress; reduction of tiredness and fatigue; regeneration of the reduced form of vitamin E. Vitamin C increases iron absorption.
Vitamin B_9_	8	Folate contributes to maternal tissue growth during pregnancy, normal amino acid synthesis, blood formation, homocysteine metabolism, psychological function, function of the immune system, reduction of tiredness and fatigue. Folate has a role in the process of cell division.
Iron	7	Iron contributes to normal cognitive function, energy-yielding metabolism, formation of red blood cells and hemoglobin, oxygen transport in the body, function of the immune system, reduction of tiredness and fatigue. Iron has a role in the process of cell division.
Copper	8	Copper contributes to maintenance of normal connective tissues, energy-yielding metabolism, functioning of the nervous system, hair pigmentation, iron transport in the body, skin pigmentation, function of the immune system, protection of cells from oxidative stress.
Manganese	4	Manganese contributes to normal energy-yielding metabolism, formation of connective tissue, maintenance of normal bones, protection of cells from oxidative stress.
Zinc	18	Zinc contributes to normal acid–base metabolism, carbohydrate metabolism, cognitive function, DNA synthesis, fertility and reproduction, macronutrient metabolism, metabolism of fatty acids, metabolism of vitamin A, protein synthesis. Zinc contributes to the maintenance of normal bones, hair, nails, skin, testosterone levels in the blood, vision, function of the immune system. Zinc contributes to the protection of cells from oxidative stress. Zinc has a role in the process of cell division.
Calcium	8	Calcium contributes to normal blood clotting, energy-yielding metabolism, muscle function, neurotransmission, function of digestive enzymes. Calcium is needed for the maintenance of normal bones and teeth. Calcium has a role in the process of cell division and specialization.
Magnesium	10	Magnesium contributes to reduction of tiredness and fatigue, electrolyte balance, normal energy-yielding metabolism, functioning of the nervous system, muscle function, protein synthesis, psychological function, maintenance of normal bones and teeth. Magnesium has a role in the process of cell division.

**Table 2:** Use conditions for health claims related to vitamin C, vitamin B_9_, iron, copper, manganese, zinc, calcium, and magnesium in the food supplement samples according to the European regulation [41,42].

Micronutrients	NRV	Required Amount to Use the Claim (15% NRV)
Vitamin C	80 mg	12 mg/100 g
Vitamin B_9_	200 µg	30 µg/100 g
Fe	14 mg	2.1 mg/100 g
Cu	1 mg	0.15 mg/100 g
Mn	2 mg	0.3 mg/100 g
Zn	10 mg	1.5 mg/100 g
Ca	800 mg	120 mg/100 g
Mg	375 mg	56.25 mg/100 g

NRV = Nutrient Reference Values.

**Table 3:** Assessment of the tolerances established for vitamin C in the food supplement samples pursuant to the European Guidance document [43].

Sample	Analytical Value (mg/sachet)	Declared Value (mg/sachet)	Range of Tolerances (RT) (mg/sachet)
S1	127.385 ± 6.179 ^a^ 111.804 ± 7.347 ^a^ $\overset{-}{x}$ **= 119.595 ± 11.017**	110	87.600–165.600
S4	217.856 ± 7.307 ^a^ 233.815 ± 7.451 ^a^ $\overset{-}{x}$ **= 225.836 ± 11.285**	225	179.600–338.100

In each column, ^a^ means statistically significant differences (*p* < 0.05) compared through Tukey’s HSD Test.

**Table 4:** Assessment of the tolerances established for vitamin C in the food supplement samples [6] pursuant to the European Guidance document [43].

Sample	Analytical Value Range (mg/sachet)	Declared Value (mg/sachet)	Range of Tolerances (RT) (mg/sachet)
S1	499.63–519.47	500	399.60–750.60
S2	399.12–418.06	400	390.03–600.60
S3	426.27–432.77	400	396.25–600.60
S4	211.60–229.78	200	159.60–300.60

**Table 5:** Mean values of the micro- and macro-elements contents analyzed in food supplement samples. Results expressed as mg/sachet.

	Fe	Cu	Mn	Zn	Ca	Mg
**S1 sample**						
Batch 1	4.856 ± 0.017 ^a^	0.954 ± 0.003 ^a^	0.070 ± 0.007 ^a^	5.205 ± 0.019 ^a^	393.016 ± 5.528 ^a^	182.486 ± 3.213 ^a^
Batch 2	4.864 ± 0.021 ^a^	0.931 ± 0.004 ^a^	0.072 ± 0.007 ^a^	5.227 ± 0.019 ^a^	387.441 ± 3.835 ^a^	177.360 ± 2.145 ^a^
**Mean**	4.860 ± 0.005	0.942 ± 0.017	0.071 ± 0.001	5.216 ± 0.016	390.228 ± 3.942	179.923 ± 3.625
**S2 sample**						
Batch 1	0.047 ± 0.005 ^a^	0.026 ± 0.000 ^a^	0.025 ± 0.002 ^a^	0.038 ± 0.003 ^a^	1.285 ± 0.038 ^a^	0.036 ± 0.003 ^a^
Batch 2	0.049 ± 0.002 ^a^	0.024 ± 0.002 ^a^	0.025 ± 0.002 ^a^	0.039 ± 0.002 ^a^	1.396 ± 0.136 ^a^	0.039 ± 0.002 ^a^
Mean	0.048 ± 0.002	0.025 ± 0.001	0.025 ± 0.000	0.038 ± 0.000	1.340 ± 0.079	0.038 ± 0.002
**S3 sample**						
Batch 1	0.017 ± 0.000 ^a^	0.014 ± 0.001 ^a^	2.009 ± 0.130 ^a^	10.791 ± 0.524 ^a^	1.010 ± 0.100 ^a^	0.090 ± 0.006 ^a^
Batch 2	0.015 ± 0.000 ^a^	0.015 ± 0.001 ^a^	2.032 ± 0.096 ^a^	10.560 ± 0.393 ^a^	1.053 ± 0.103 ^a^	0.078 ± 0.001 ^a^
**Mean**	0.705 ± 0.066	0.014 ± 0.000	2.020 ± 0.016	10.675 ± 0.163	1.031 ± 0.030	0.084 ± 0.008
**S4 sample**						
Batch 1	6.123 ± 0.060 ^a^	0.555 ± 0.012 ^a^	0.989 ± 0.030 ^a^	6.033 ± 0.224 ^a^	200.358 ± 1.456 ^a^	153.869 ± 6.987 ^a^
Batch 2	6.153 ± 0.096 ^a^	0.550 ± 0.009 ^a^	1.000 ± 0.037 ^a^	6.011 ± 0.210 ^a^	200.213 ± 6.087 ^a^	148.602 ± 8.524 ^a^
**Mean**	6.138 ± 0.021	0.553 ± 0.003	0.995 ± 0.008	6.022 ± 0.016	200.286 ± 0.102	151.236 ± 3.724

In each column, ^a^ means statistically significant differences (*p* < 0.05) compared through Tukey’s HSD Test.

**Table 6:** Assessment of ranges of tolerance (RT) established for those minerals whose contents are available in the labeling of each selected samples according to the European Guidance document [43]. Results expressed as mg/sachet product.

Mineral	Analytical Value (mg/sachet)	Declared Value (mg/sachet)	Range of Tolerances (RT) (mg/sachet)
**Fe**			
S1 sample	4.855–4.865	5	4–7.25
S4 sample	6.117–6.159	6	4.8–8.7
**Cu**			
S1 sample	0.925–0.959	1	0.8–1.45
S4 sample	0.550–0.555	0.5	0.4–0.725
**Mn**			
S3 sample	2.004–2.036	2	1.6–2.9
S4 sample	0.987–1.003	1	0.8–1.45
**Zn**			
S1 sample	5.200–5.232	5	4–7.25
S3 sample	10.512–10.838	10	8–14.5
S4 sample	6.006–6.038	6	4.8–8.7
**Ca**			
S1 sample	386.286–394.17	400	320–580
S4 sample	200.184–200.388	200	160–290
**Mg**			
S1 sample	176.298–183.548	180	144–261
S4 sample	147.512–154.960	150	120–217.5

## Data Availability

Not applicable.
